# Acquisition of host-derived carbon in biomass of the ectomycorrhizal fungus *Pisolithus microcarpus* is correlated to fungal carbon demand and plant defences

**DOI:** 10.1093/femsec/fiad037

**Published:** 2023-03-31

**Authors:** Emiko K Stuart, Vasanth Singan, Mojgan Amirebrahimi, Hyunsoo Na, Vivian Ng, Igor V Grigoriev, Francis Martin, Ian C Anderson, Jonathan M Plett, Krista L Plett

**Affiliations:** Hawkesbury Institute for the Environment, Western Sydney University, Richmond, NSW 2753, Australia; US Department of Energy Joint Genome Institute, Lawrence Berkeley National Laboratory, Berkeley, CA 94720, USA; US Department of Energy Joint Genome Institute, Lawrence Berkeley National Laboratory, Berkeley, CA 94720, USA; US Department of Energy Joint Genome Institute, Lawrence Berkeley National Laboratory, Berkeley, CA 94720, USA; US Department of Energy Joint Genome Institute, Lawrence Berkeley National Laboratory, Berkeley, CA 94720, USA; US Department of Energy Joint Genome Institute, Lawrence Berkeley National Laboratory, Berkeley, CA 94720, USA; Department of Plant and Microbial Biology, University of California Berkeley, Berkeley, CA 94720, USA; Université de Lorraine, INRAE, Interactions Arbres/Microorganismes, F-54000 Nancy, France; Hawkesbury Institute for the Environment, Western Sydney University, Richmond, NSW 2753, Australia; Hawkesbury Institute for the Environment, Western Sydney University, Richmond, NSW 2753, Australia; Hawkesbury Institute for the Environment, Western Sydney University, Richmond, NSW 2753, Australia; NSW Department of Primary Industries, Elizabeth Macarthur Agricultural Institute, Menangle, NSW 2568, Australia

**Keywords:** carbon, ectomycorrhizal symbiosis, *Pisolithus microcarpus*, stable isotopes, transcriptomics

## Abstract

Ectomycorrhizal (ECM) fungi are key players in forest carbon (C) sequestration, receiving a substantial proportion of photosynthetic C from their forest tree hosts in exchange for plant growth-limiting soil nutrients. However, it remains unknown whether the fungus or plant controls the quantum of C in this exchange, nor what mechanisms are involved. Here, we aimed to identify physiological and genetic properties of both partners that influence ECM C transfer. Using a microcosm system, stable isotope tracing, and transcriptomics, we quantified plant-to-fungus C transfer between the host plant *Eucalyptus grandis* and nine isolates of the ECM fungus *Pisolithus microcarpus* that range in their mycorrhization potential and investigated fungal growth characteristics and plant and fungal genes that correlated with C acquisition. We found that C acquisition by *P. microcarpus* correlated positively with both fungal biomass production and the expression of a subset of fungal C metabolism genes. In the plant, C transfer was not positively correlated to the number of colonized root tips, but rather to the expression of defence- and stress-related genes. These findings suggest that C acquisition by ECM fungi involves individual fungal demand for C and defence responses of the host against C drain.

## Teaser

Mutualistic ectomycorrhizal fungi are able to sequester carbon based on their growth characteristics and on the degree to which they evade host immune defence responses.

## Introduction

Ectomycorrhizal (ECM) fungi colonize the roots of most trees in temperate and boreal forests and, as these ecosystems make up the majority of the global terrestrial carbon (C) sink, are crucial to C cycling (Taylor et al. 2000, Martin et al. 2001, Pan et al. 2011, Averill et al. 2014, Wu et al. 2022). In nutrient-limited forests, ECM fungi colonize receptive plant roots and provide plants with nutrients from surrounding soil. These nutrients are released into a specialized colonization structure in the root tip called the Hartig net, where ECM fungi obtain up to 30% of photosynthetically fixed plant C (Leake et al. 2004, Hobbie 2006, Ekblad et al. 2013). While ECM fungi have specialized abilities to acquire plant growth-limiting nutrients such as nitrogen (N) and phosphorus (P) from soil organic and inorganic matter, and improve water uptake, they remain dependent on root-exuded C due to their limited capacities to metabolize complex carbohydrates (Nehls et al. 2010, Lindahl and Tunlid 2015, Liu et al. 2020). The direct access of ECM fungi to this C entails a competitive advantage over other soilborne microbes and therefore alters soil respiration and may improve belowground C storage (Gadgil and Gadgil 1971, 1975, Averill et al. 2014, Averill and Hawkes 2016, Gorka et al. 2019).

Despite the importance of ECM symbiosis in C cycling, mechanisms controlling host-to-fungus C transfer and greater ecosystem impacts of ECM-associated C cycling are not well understood (reviewed in Stuart and Plett 2020). Some studies suggest that ECM host plants control C allocation through reciprocal rewards- or sanctions-based mechanisms, whereby plants reward more C to fungi that provide more growth-limiting nutrients (Kytöviita 2005, Kiers et al. 2011, Casieri et al. 2013, Bogar et al. 2019). Alternatively, plants may increase belowground C allocation in response to soil nutrient limitation to encourage greater soil nutrient supply by the fungi (Hobbie 2006, Nehls et al. 2010, Näsholm et al. 2013). Others suggest that belowground C allocation may operate based on a source-sink mechanism where nutrient transfer is influenced by fungal C demand. This sink may be created by increases to fungal biomass production or allocation of C towards assimilating N (Wallander 1995, Corrêa et al. 2008, 2012, Lemoine et al. 2013). Moreover, host defence responses may play a role in controlling C loss via attack on, and senescence of, roots colonized by uncooperative fungi (Kiers and Denison 2008, Garcia et al. 2015, Hortal et al. 2017, Bogar et al. 2019). More research is needed to understand these defence mechanisms.

Understanding of ECM C transfer at the molecular level is limited (Durall et al. 1994, Pumpanen et al. 2009, Heinonsalo et al. 2010, Pickles et al. 2016), although transporters and enzymes that may facilitate sugar transfer and catabolism at the plant–fungal interface have been found to be upregulated in colonized plant roots (Wright et al. 2000, Nehls et al. 2010, Plett et al. 2015a, b, Bouffaud et al. 2020, Ruytinx et al. 2021, Tang et al. 2021). While some host-derived C may be lost via ECM fungal respiration or exudation, a substantial amount is sequestered by rapid conversion into various storage compounds including trehalose, mannitol, and glycogen (Martin et al. 1987, López et al. 2007, Nehls et al. 2007, Hagenbo et al. 2019). Such a sequestration mechanism suggests that the ability of ECM fungi to produce biomass should be correlated to their acquisition of host-derived C. Leake et al. (2001) noted that increases in the hyphal density of *Paxillus involutus* occurred in litter patches that contained greater amounts of C derived from the host plant *Pinus sylvestris*, indicating a link between fungal biomass production and C acquisition. However, there is a current lack of studies quantifying the correlation between fungal biomass production and amounts of host-derived C acquired by a greater range of ECM fungi, partly due to the difficulties of measuring fungal biomass and C allocation in field experiments (Hobbie and Argerer 2010, Wallander et al. 2013).

There is considerable genetic diversity amongst ECM fungal species leading to potential interspecies variation in the genetic mechanisms supporting C acquisition from their hosts (Read and Perez-Moreno 2003, Rinaldi et al. 2008, Tedersoo and Smith 2013, Martin et al. 2016, Tedersoo and Brundrett 2017). For example, C starvation, rather than C availability, was shown to induce expression of fungal monosaccharide transporter genes by *Laccaria bicolor* (Fajardo López et al. 2008). In *Amanita muscaria*, the sugar transporter AmMst1 is upregulated under increased C sugar concentrations (Nehls et al. 2001). While these differences in C exchange and metabolism have been found to occur between distantly related fungal species, more recent work would suggest that there can be distinct variation within one ECM fungal species (Hortal et al. 2017, Plett et al. 2021). This suggests that understanding of this function should be improved to a finer scale than current genus-level knowledge.

Here, we investigated the effects of intraspecies transcriptomic and physiological variability on C exchange between nine isolates of *Pisolithus microcarpus* that ranged in mycorrhization potential, and their host *Eucalyptus grandis*. The amount of C transferred from host to fungus and incorporated into fungal biomass was measured via ^13^C stable isotope labelling using a microcosm system, and growth differences between the fungal isolates were examined to determine whether growth-related traits correlated with the amounts of C acquired. Gene expression in the fungi and in the host plant were analysed to find expression patterns that may correlate to C acquisition by the fungi from the plant. Based on the literature discussed above, we hypothesized that there would be significant intraspecies variation in the amounts of host-derived C in fungal biomass, and that these amounts would positively correlate with fungal growth and colonization rate. We also hypothesized that the expressions of both plant and fungal genes relating to C transport and metabolism, including both catabolism and biosynthesis, would correlate with amounts of host-derived C, and that decreased gene expression of plant defence mechanisms would allow for greater C acquisition by the fungi.

## Experimental procedures

### Biological material and experimental microcosm setup

Nine *P. microcarpus* isolates were originally isolated from various locations across Australia (scientific license number S13146; Supplementary Table S1; Keniry 2015) or New Caledonia (Jourand et al. 2010). The fungal isolates were selected as they represent a gradient of host mycorrhization potential (e.g. Plett et al. 2015a). Pure cultures of these fungal isolates were maintained on modified Melin Norkrans (MMN) agar plates (0.5 g/L (NH_4_)_2_HPO_4_, 0.3 g/L KH_2_PO_4_, 0.14 g/L MgSO_4_.7H_2_O, 10 g/L glucose, 1 mL/L of CaCl_2_ 5% w/v stock solution, 1 mL/L of NaCl 2.5% w/v stock solution, 1 mL/L of ZnSO_4_ 0.3% w/v stock solution, 133 μL/L of thiamine 0.1% w/v stock solution, 1 mL/L of citric acid + Fe EDTA 1.25% w/v stock solution and 13 g/L agar in de-ionized water, pH 5.5). The plates were incubated in a growth chamber in the dark at 25°C (ICP 800, Memmert, Schwabach, Bavaria, Germany).

Phylogenetic analysis of the *P. microcarpus* isolates was conducted with the online tool Phylogeny.fr (Dereeper et al. 2008). ITS sequences were obtained from the National Center for Biotechnology Information (NCBI) Nucleotide database (https://www.ncbi.nlm.nih.gov/nuccore) or sequenced at the Hawkesbury Institute for the Environment (Western Sydney University, Richmond, New South Wales, Australia; Supplementary Table S2). ITS sequences were aligned using MUSCLE 3.8.31 and poorly aligned positions and divergent regions were eliminated using Gblocks 0.91b. The phylogenetic tree was constructed using the maximum likelihood method (PhyML3.1/3.0 aLRT) and drawn with TreeDyn 198.3.

*Eucalyptus grandis* seeds (seedlot no. 21 068) were obtained from the Commonwealth Scientific and Industrial Research Organisation (CSIRO, Clayton, Victoria, Australia) Australian Tree Seed Centre. *Eucalyptus grandis* seeds were surface sterilized with 30% hydrogen peroxide for 10 min and germinated on 1% w/v water agar in a plant growth chamber (TRISL-495-1-SD, Thermoline Scientific, Smithfield, New South Wales, Australia) under a 16-h light cycle at 25°C. After 4 weeks of growth, the seedlings were transferred, three seedlings per plate and on top of a sterile cellophane membrane, to fresh, half-strength (½) MMN agar plates containing 0.1% w/v glucose. The plates were incubated for another 4 weeks under the same conditions. Meanwhile, small squares (0.5 × 0.5 cm) of each *P. microcarpus* isolate were cut from the growing edge of the fungal cultures grown on 1× MMN cultures, and were transferred to fresh ‘low glucose’ ½ MMN agar plates, made with the same recipe as ½ MMN but only containing 0.01% w/v glucose, on top of a sterile cellophane membrane. Eight cultures per isolate were prepared. The plates were incubated in a growth chamber (ICP 800) in the dark at 25ºC.

After 2 weeks of fungal growth on the low glucose ½ MMN, individual *E. grandis* seedlings were carefully transferred onto the edge of growing *P. microcarpus* mycelium. In total, five plant + fungus microcosms (‘ECM’ condition) per *P. microcarpus* isolate were set up. As controls, three microcosms per isolate with fungi only [free-living mycelium (FLM) control] were also maintained, and three *E. grandis* seedlings were transferred onto low glucose ½ MMN as plant only controls. The plates were sealed with one-third micropore tape at the top of the plate, to allow for free gas exchange, and two-thirds electrician’s tape. The plates were placed in a plant growth chamber (GC20 BDAF, Bio Chambers Incorporated, Winnipeg, Manitoba, Canada) at a 45°C angle and incubated under a 16 h photoperiod, light intensity of 500 μmol m^−2^ s^−1^, 25°C/18°C day/night temperature, 70% relative humidity, and ambient CO_2_ (400 ppm) for 2 weeks, at which the fungus would have fully colonized the root system and established nutrient trading as previously shown (Hortal et al. 2017). Within the 2 week co-culture, at 9 days post fungal contact, two holes were opened on the front of each plate using a soldering iron and the holes were covered with micropore tape to further increase gas permeability into the plates. The plates were placed in a 175 L airtight polycarbonate chamber with an air circulation fan and a gas injection port inside the plant growth chamber (for more details, see Hortal et al. 2017). The tank was injected with 12 mL of 99 atom % ^13^CO_2_ (Sigma-Aldrich, St Louis, MI, USA) and the plates were left in the tank for 5 hours under circulating air. Following this, the tank was opened and allowed to equilibrate back to ambient levels of ^13^CO_2_. At day 11 of co-culture, the microcosms were once again placed in the labelling chamber and another 12 mL of 99 atom % ^13^CO_2_ was injected into the tank and the plates were again left in the tank for 5 hours under circulating air followed by return to ambient ^13^CO_2_ conditions until harvest. Free-living mycelium and plant only control plates were grown and incubated under the same conditions.

For the measurement of fungal biomass, four to five more replicates per isolate of the FLM control plates were set up and treated in the same way as described above, except that they did not undergo ^13^C labelling.

### ^13^C stable isotope analysis

Three days after the second injection of ^13^CO_2_, following sampling for RNA-seq analysis (see below), all of the remaining fungal extra-radical mycelium (ERM) and plant leaves from the low glucose ½ MMN plates (four to five replicates of the ECM symbiosis plates and three replicates of the FLM fungus-only plates) were harvested, oven-dried at 40ºC and ground to a fine powder. ^13^C atom % values were measured by running about 1 mg of ground fungal tissue or about 2 mg of ground plant tissue on an elemental analyser and isotope ratio mass spectrometer (UC Davis Stable Isotope Facility, Davis, CA, USA). Outlier ^13^C atom % values were identified using the interquartile range test and removed from the data set. ^13^C atom % values were converted to amounts of C transferred from the plant host to the fungus via symbiosis and retained, as respired or exuded C was not accounted for, using the calculations below based on methods previously described (Tomm et al. 1994, He et al. 2009, Plett et al. 2020). Herein, ‘C acquisition data’ refer to these C values.


\begin{eqnarray*} {\rm{\ \% }}{{\rm{C}}}_{{\rm{symbiosis}}} = {\rm{\ }}\frac{{\left( {13{{\rm{C}}}_{{\rm{ERM}}}{\rm{\ }} - {\rm{\ }}13{{\rm{C}}}_{{\rm{FLM}}}} \right)}}{{\left( {13{{\rm{C}}}_{{\rm{leaf}}}{\rm{\ }} - {\rm{\ }}13{{\rm{C}}}_{{\rm{FLM}}}} \right)}}{\rm{\ }} \times {\rm{\ }}100, \end{eqnarray*}

\begin{eqnarray*} {{\rm{C}}}_{{\rm{symbiosis}}} = {\rm{\ \% }}{{\rm{C}}}_{{\rm{symbiosis}}}{\rm{\ }} \times {\rm{\% }}{{\rm{C}}}_{{\rm{fungal}}}{\rm{\ }} \times {\rm{\ fungal\ biomass}}, \end{eqnarray*}

where %C_symbiosis_ is the percentage of fungal C derived from the plant host, ^13^C_ERM_ is the ^13^C atom % of the fungal ERM of the symbiosis plates, ^13^C_FLM_ is the average ^13^C atom % of the fungal mycelium of the fungus only plates, ^13^C_leaf_ is the ^13^C atom % of the leaves of the symbiosis plates, C_symbiosis_ is the amount of fungal C derived from the plant host disregarding any subsequently respired or exuded C, %C_fungal_ is the average percentage of total C in the dried fungal mycelium, and fungal biomass is the average mass of the dried fungal mycelium of the fungus only plates.

### Fungal growth parameters

The following parameters were measured as they can be considered as proxies for fungal growth.

Colonization percentage was calculated from four to five replicates per fungal isolate from the ECM symbiosis plates. This calculation was the number of lateral plant root tips colonized by the fungus (i.e. exhibiting short, thickened appearance and the presence of a fungal mantle over the root) divided by the total lateral plant root tips in contact with fungal mycelium.

For the measurement of Hartig net depth and mantle thickness, colonized plant root tips from each isolate treatment were harvested from the ECM symbiosis plates, fixed in 4% w/v paraformaldehyde and stored at 4ºC for at least 24 h. The root tips were then embedded in 6% w/v agarose and refrigerated overnight. For each treatment, cross-sections of 30 µm thickness from three independent biological replicates were cut using a Leica VT1200 vibratome (Leica Microsystems, Mt Waverley, Victoria, Australia), stained with DAPI stain and imaged using an inverted Leica SP6 confocal microscope (Leica Microsystems). The Hartig net depths and thicknesses of the fungal mantles surrounding the plant root tips were measured on four to seven sections per cross-section using the ImageJ processing program (National Institutes of Health, Bethesda, MD, USA; Schneider et al. 2012).

Growth rate, biomass, and hyphal density of the fungal isolates were determined as follows from four to five replicates per isolate of the fungus only plates, rather than from symbiosis plates, due to the difficulty in fully separating fungal hyphae from plant roots. After 2 and 4 weeks of growth on the low glucose media, the perimeters of the fungal colonies were marked, the colonies were imaged and areas of the fungal colonies and of the original agar blocks determined using ImageJ. The mycelium was harvested, oven-dried at 40ºC and weighed to determine the biomass values. Average radial mycelial growth, calculated as the area of radial growth per day from the second week of growth, or ‘plant contact’, to the fourth week of growth, or ‘harvest’, was used as a proxy for growth rate during symbiosis with the plant host. Hyphal density was calculated as the fungal biomass per unit area of the fungal colony at harvest.

### RNA extraction and RNA-seq analysis

After 4 weeks of growth on the low glucose ½ MMN, a strip of fungal ERM (including the oldest and newest part of the colony) and plant roots in contact with the fungus (excluding tap roots) were harvested from three replicates of symbiosis or plant only control low glucose plates and immediately frozen at −80°C. Total RNA (from the combined fungal ERM and plant roots for three replicates per treatment) was extracted using the ISOLATE II miRNA kit (Bioline, London, UK). The large RNA fraction from these extractions was sequenced at the Joint Genome Institute (JGI) as detailed in Plett et al. (2020).

Prior to normalization of the count data, genes that were not expressed under any isolate condition (having an average read count within each condition of <10 mapped reads for *E. grandis* and <5 for *P. microcarpus*) were removed from the data. This resulted in sets of 24 615 *E. grandis* transcripts and 12 959 *P. microcarpus* transcripts as ‘expressed’ under at least one condition. The DESeq2 R package (v. 3.6.1) was used to normalize the count data. Functional annotations for the following genomes were obtained from the JGI Genome Portal: *E. grandis* genome v. 2.0 (Myburg et al. 2014; https://phytozome-next.jgi.doe.gov/info/Egrandis_v2_0), *P. microcarpus* 441 v 1.0 (Kohler et al. 2015; https://mycocosm.jgi.doe.gov/Pismi2/Pismi2.home.html).

### Heatmap visualization

*Eucalyptus grandis* C transporter genes, *P. microcarpus* C transporter genes, and *P. microcarpus* C metabolism genes were obtained from Hortal et al. (2017) and Plett et al. (2021). For heatmap visualization, fold change in gene expression for each isolate was calculated by dividing the DESeq2-normalized gene expression values by the average expression values of all of the isolates (i.e. the baseline expression level), and log_2_-transformed. Heatmaps were generated using the online tool Morpheus (Broad Institute, Cambridge, MA, USA, https://software.broadinstitute.org/morpheus). Hierarchical clustering for C transporter gene heatmaps was performed using the Euclidean distance measure. Carbon transporter and C metabolism genes of interest were identified as genes for which Wil3 had the highest or lowest level of expression of the fungal isolates. This is because Wil3 received significantly greater amounts of ^13^C from the host than most other fungal isolates, so genes with comparatively higher or lower levels of expression in Wil3 may be related to fungal C acquisition.

### Pearson’s correlation analysis of C acquisition data

The DESeq2-normalized count data for the expressed *E. grandis* and *P. microcarpus* transcripts were correlated to the amounts of host-derived C acquired by the fungi according to the method described by Baute et al. (2015). Briefly, Pearson’s correlation coefficients between the averaged C acquisition data and the count data, from the three replicates on which transcriptomic analysis was performed, for each transcript were calculated. Transcripts with Pearson’s correlation coefficients in the top percentile (q_0.99_, i.e. most positively correlated transcripts) and bottom percentile (q_0.01_, i.e. most negatively correlated transcripts) of correlation values were identified. All Pearson’s correlation coefficients for the identified transcripts were statistically significant according to the Pearson Correlation Table of Critical Values.

The functional annotations of these sets of positively and negatively correlated transcripts were searched to identify genes involved in C metabolism (biosynthesis and catabolism), defence/disease resistance, growth/cell cycle regulation, host–fungus interaction, signal transduction, stress response, transcription regulation, or transport, as these categories of genes may play a role in C acquisition beyond the C transporter and metabolism genes used for heatmap visualization.

### Statistical analyses

ANOVA and Tukey’s Honest Significant Different (HSD) test for *post-hoc* analysis were performed on the C acquisition, growth, and colonization data using the R stats package (v. 3.6.1). Principal component analysis (PCA) of log-transformed symbiotic C acquisition and fungal growth characteristics data was performed using the prcomp function in R. Correlation analyses were conducted in Microsoft Excel (v. 16) to examine the association between the amounts of host-derived C acquired by the fungal isolates and their growth parameters. For correlation between C acquisition and colonisation rate, data from paired samples were used. For correlation between C acquired and other growth parameters (biomass, hyphal density, Hartig net depth, mantle thickness, and growth rate), averaged C acquisition data and individual growth parameter data were used as these groups of datasets were obtained from nonpaired samples.

## Results

### Growth characteristics and acquisition of host-derived C varies significantly between *P. microcarpus* isolates

**Figure 1.**
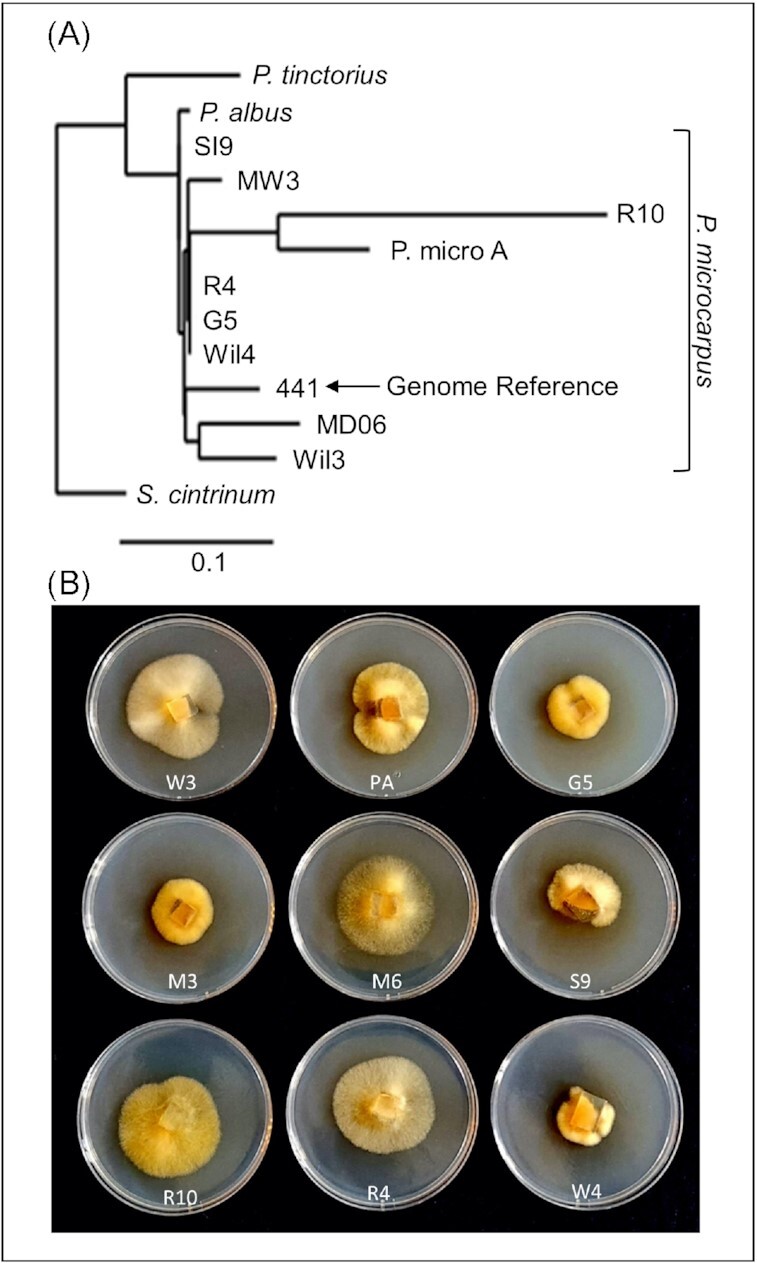
Phylogenetic relationship and morphological variation of *P. microcarpus*. (A) ITS-based phylogenetic tree of the studied *P. microcarpus* isolates and the *P. microcarpus* reference isolate 441 v 1.0. ITS sequences for *P. albus, P. tinctorius*, and *Scleroderma citrinum* are used as outgroups. Phylogenetic analysis and tree construction were performed with Phylogeny.fr using ‘one click’ mode. Branch length represents sequence divergence (scale bar represents 0.1 substitutions per site). (B) *Pisolithus microcarpus* isolates used in this manuscript grown on low glucose ½ modified Melin Norkrans agar plates. Each isolate is annotated in the figure as follows: W3—Wil3, PA—P. micro A, G5—G5, M3—MW3, M6—MD06, S9—SI9, R10—R10, R4—R4, W4—Wil4.

Acquisition of host-derived C was investigated using nine fungal isolates. A phylogenetic tree was constructed and all isolates were found to group with *P. microcarpus* reference isolate 441, distinct from the closely related *P. albus* and the more basal species *P. tinctorius* (Fig. 1A). The morphology of the fungal colonies grown *in vitro* on low glucose plates varied notably, ranging from sparse, far-reaching mycelia to denser and shorter mycelia (Fig. 1B).

**Figure 2.**
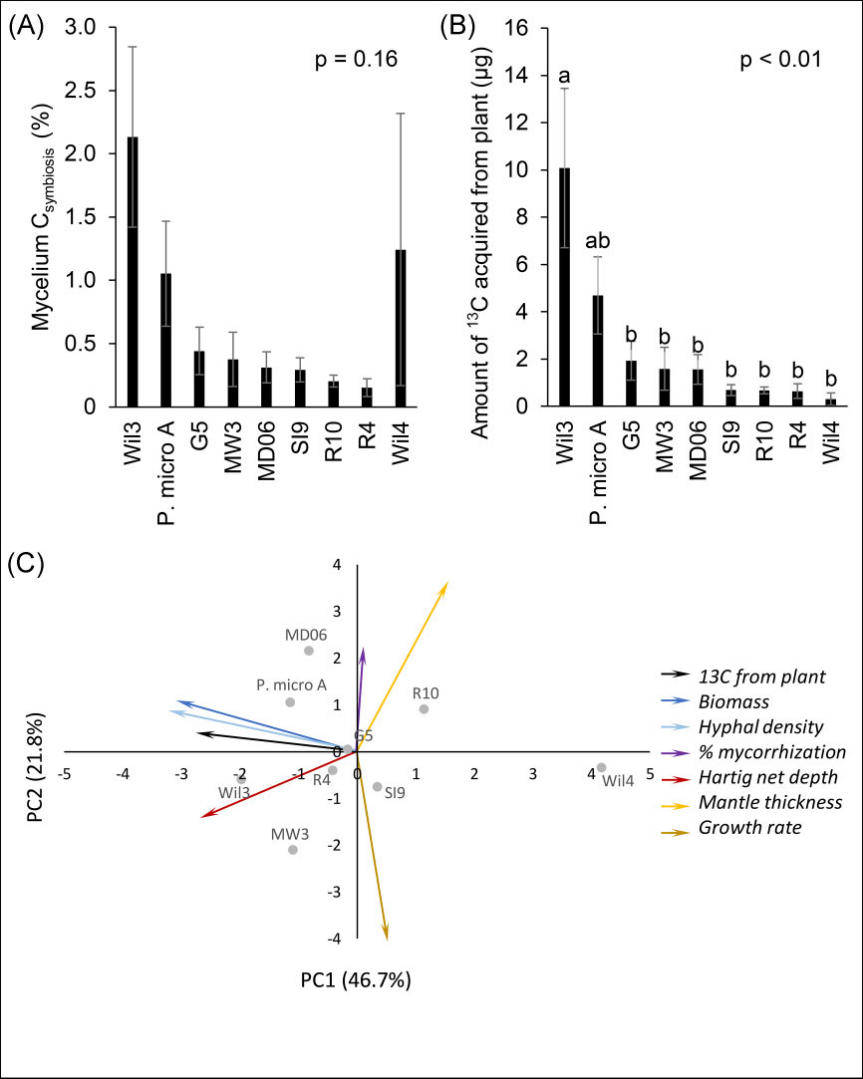
*Pisolithus microcarpus* shows significant intraspecies variation in acquisition of host-derived carbon (C) that is related to fungal biomass and hyphal density. (A) Percentage of mycelium C acquired by *P. microcarpus* isolates from *E. grandis* host. (B) Amount of ^13^C acquired by *P. microcarpus* isolates from *E. grandis* host. (C) PCA showing variation between fungal isolates in terms of their amount of symbiotic C acquisition (black arrow) and fungal growth characteristics (coloured arrows). Different letters indicate statistically significant (*P* < .05) differences as determined via the Tukey’s HSD test. Error bars indicate standard error.

Symbiotic C acquisition by each *P. microcarpus* isolate from its host was determined through ^13^C stable isotope analysis of the fungal ERM. Differences between the isolates in the percentage of ERM C derived from symbiosis were not statistically significant, due to the large variation in the Wil4 measurements (Fig. 2A). Statistical significance was observed when Wil4 was removed from the data (*P* < .01). While the percentage of mycelial C derived from symbiosis gives a proportional measure of host C present in ERM tissue per unit mass, it did not represent the total amount of C estimated to be transferred to fungal tissues. Therefore, the total amount of host C acquired by symbiosis and retained in hyphal tissues (i.e. did not account for exuded or respired C) was calculated using the C content and overall biomass estimates of the fungal colonies. There was significant variation in the amount of biomass produced by the isolates *in vitro* and in the percentage of C of the fungal mycelia (Table 1), amplifying the differences in C acquisition amongst the *P. microcarpus* isolates such that the amounts of host-acquired C differed significantly between the isolates (Fig. 2B). Wil4, while containing a high percentage of C from symbiosis in some replicates, had the smallest amount of biomass of the fungi considered, and thus showed the lowest amount of total host C acquired. PCA was conducted to find relationships between symbiotic C acquisition by the fungus and measured fungal growth characteristics (Fig. 2C). PCA 1 and 2 together explained 68.6% of the variance. Along the PC1 axis, the amount of symbiotic C acquired by the fungal isolates was most closely related to biomass, hyphal density and Hartig net depth.

**Table 1.** Growth characteristics of *P. microcarpus* isolates.

Isolate	Biomass (mg)	Mycelium C (%)	Hyphal density (mg/cm^2^)	Hartig net depth (µm)	Mantle thickness (µm)	Radial mycelial growth rate (% area/day)
Wil3	1.20 ± 0.08^a^	38.92 ± 0.57^ab^	0.07 ± 0.01^a^	14.78 ± 0.57^a^	35.54 ± 5.18^abcd^	15.56 ± 2.47^ab^
P. micro A	1.18 ± 0.07^a^	38.31 ± 0.60^b^	0.06 ± 0.00^a^	9.90 ± 0.76^b^	42.08 ± 7.50^abcd^	8.89 ± 1.50^bc^
SI9	0.62 ± 0.23^ab^	37.93 ± 0.17^b^	0.04 ± 0.02^ab^	6.16 ± 0.50^e^	21.29 ± 1.16^d^	13.16 ± 1.56^ab^
MD06	1.30 ± 0.14^a^	37.74 ± 0.44^b^	0.07 ± 0.01^a^	9.64 ± 0.68^bc^	57.26 ± 3.39^ab^	4.24 ± 0.68^c^
R4	1.10 ± 0.15^a^	37.54 ± 0.68^b^	0.03 ± 0.00^ab^	15.99 ± 0.82^a^	33.28 ± 7.78^abcd^	8.86 ± 0.57^bc^
G5	1.15 ± 0.20^a^	37.84 ± 0.30^b^	0.05 ± 0.01^ab^	7.08 ± 0.45^bd^	49.24 ± 5.53^abcd^	11.13 ± 1.22^bc^
MW3	0.97 ± 0.11^a^	41.93 ± 1.33^a^	0.05 ± 0.01^ab^	16.62 ± 0.60^a^	24.21 ± 0.95^cd^	21.10 ± 0.77^a^
Wil4	0.08 ± 0.05^b^	39.49 ± 0.75^ab^	0.01 ± 0.01^b^	4.52 ± 0.57^e^	62.90 ± 6.90^a^	14.75 ± 2.92^ab^
R10	0.90 ± 0.13^a^	36.86 ± 0.20^b^	0.03 ± 0.00^ab^	6.72 ± 0.53^cde^	59.52 ± 8.52^abc^	13.81 ± 1.50^ab^
*P*-value	<.001***	<.01**	<.01**	<.001***	<.001***	<.001***

Values are displayed as mean ± SE. *P*-values for each characteristic were determined by one-way ANOVA. Different letters indicate statistically significant (*P* < .05) differences as determined via the Tukey’s HSD test.

### Acquisition of host-derived C by *P. microcarpus* is significantly correlated to biomass accumulation, hyphal density, and hartig net depth

**Figure 3.**
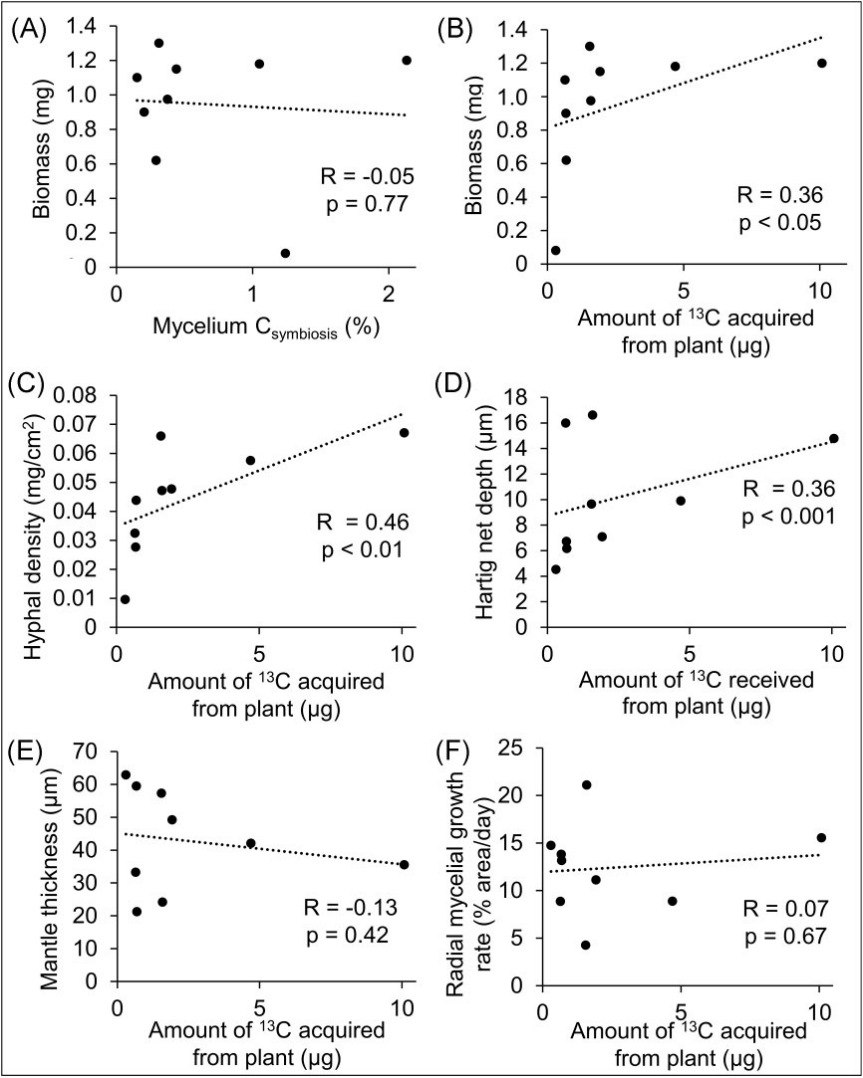
Acquisition of host-derived carbon (C) by *P. microcarpus* correlates to fungal biomass production, hyphal density, and Hartig net depth. (A) Correlation of percentage of fungal mycelium C acquired from host with fungal biomass production. (B–F) Correlation of amount of ^13^C acquired from *E. grandis* host with fungal biomass production (B), hyphal density (C), Hartig net depth (D), mantle thickness (E), and radial mycelial growth rate (F). Dots represent the averaged data of the *P. microcarpus* isolates. Dotted lines indicate lines of best fit.

As predicted by the PCA, while fungal biomass did not correlate with the percentage of mycelial C derived from symbiosis, it did positively correlate with the amount of host-derived C (Fig. 3A and B). Similarly, the amount of host-derived C correlated significantly with the density of fungal hyphae (Fig. 3C) and Hartig net depth (Fig. 3D), but did not significantly correlate with the thickness of the fungal mantle surrounding plant root tips (Fig. 3E) nor with radial mycelial growth rate (Fig. 3F).

### Root colonization rate negatively correlates with host C acquisition by *P. microcarpus*

**Figure 4.**
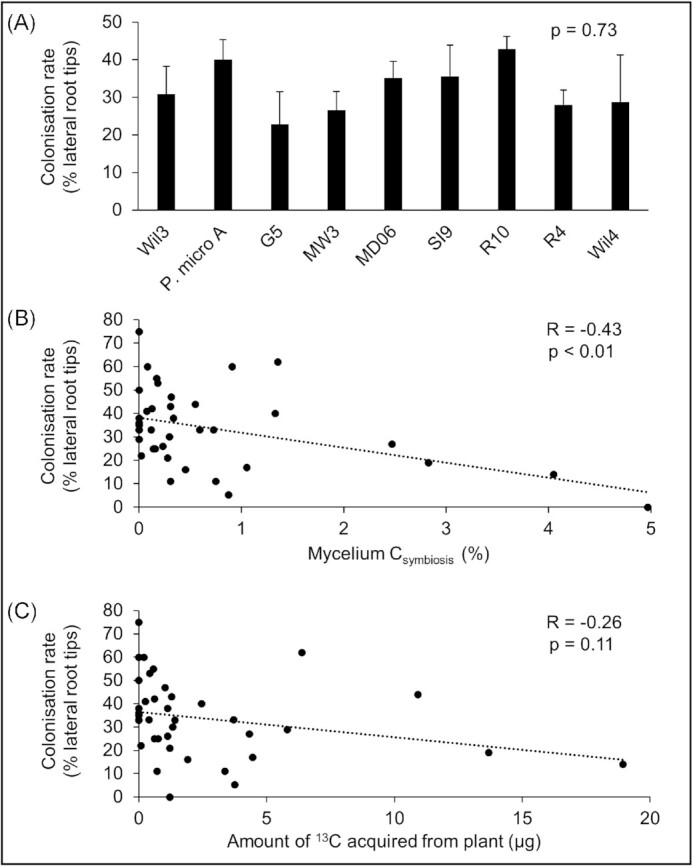
Acquisition of host-derived carbon (C) by *P. microcarpus* negatively correlates with plant root colonization. (A) Percentages of *E. grandis* lateral roots colonised by *P. microcarpus* isolates. (B) Correlation of percentage of fungal mycelium C acquired from host with plant root colonization rate. (C) Correlation of amount of ^13^C acquired from host with plant root colonization rate. Different letters indicate statistically significant (*P* < .05) differences as determined via the Tukey’s HSD test. Each dot represents the paired C and colonization data for one replicate. Dotted lines indicate lines of best fit.

Plant root colonization rate did not significantly differ between the fungal isolates used in this experiment (Fig. 4A). Correlation analyses were performed to determine whether plant root colonization rate correlated with the percentage or amount of host-derived C acquired by the fungi. Colonization rate was significantly, negatively correlated with the percentage of symbiotic C retained in the fungal mycelium (*P* < .05; Fig. 4B). Colonization rate had a weakly negative correlation with the amount of host-derived C, although this was not statistically significant (*P* = .11; Fig. 4C).

### *Pisolithus microcarpus* C acquisition is more related to expression of C metabolism genes than C transporter genes

**Figure 5.**
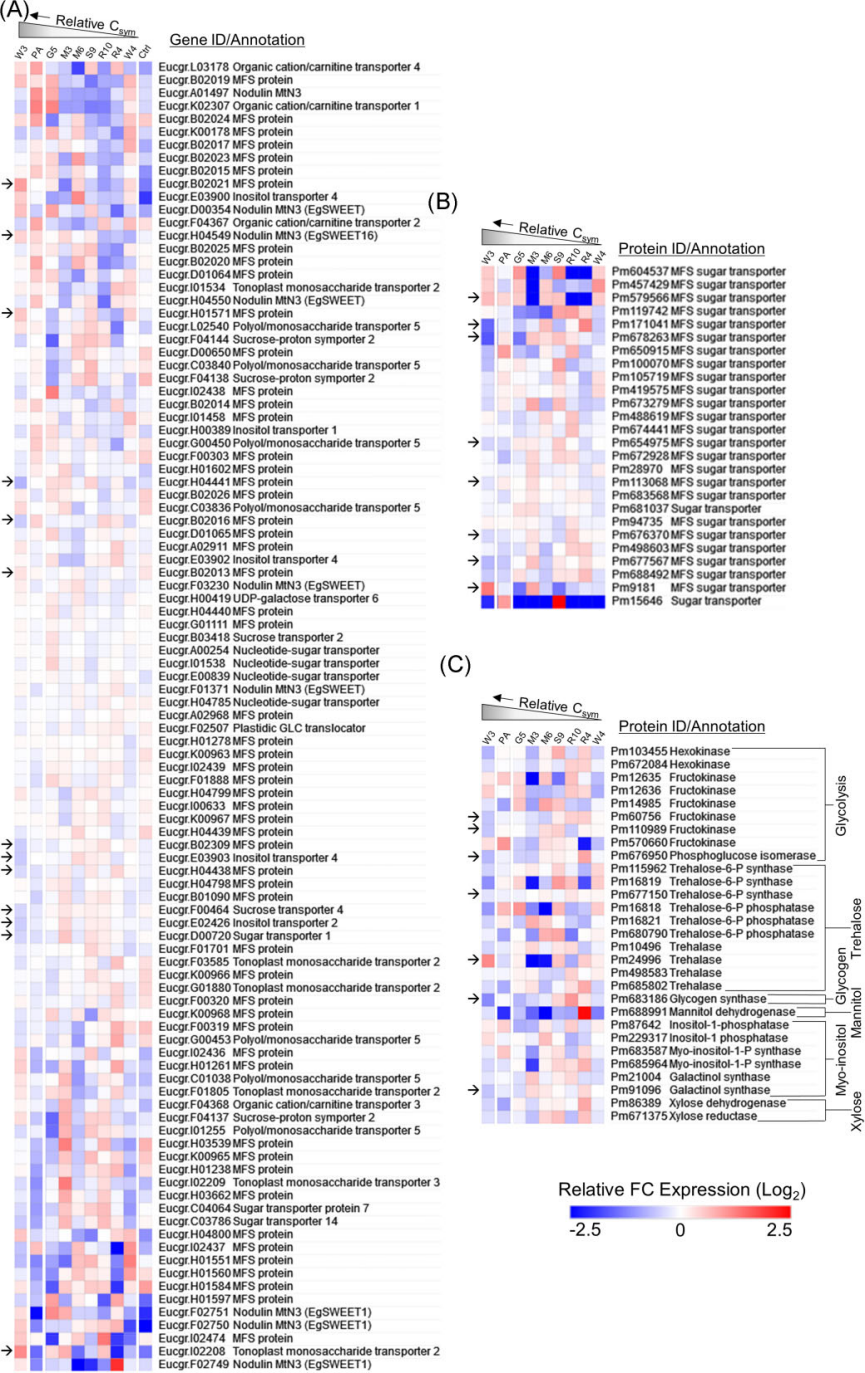
Host-derived carbon (C) acquisition is largely unrelated to expression of plant and fungal genes for C transport and metabolism. Heatmaps of DESeq2-normalized and log_2_-transformed expression values of (A) *E. grandis* C transporter genes, (B) *P. microcarpus* C transporter genes, and (C) *P. microcarpus* C metabolism genes. Heatmaps were generated using Morpheus online tool. Hierarchical clustering for C transporter gene heatmaps was performed using the Euclidean distance measure. Carbon metabolism genes were grouped by C metabolism pathway, as indicated to the right of the heatmap. Isolates labelled as ‘high C’ and ‘low C’ had statistically significant differences in amounts of acquired host-derived C, as determined by ANOVA. Arrows indicate genes of interest that are referenced in the text and are identified as genes for which Wil3 had the highest or lowest level of expression out of the fungal isolates. Columns labelled as ‘Ctrl’ represent uninoculated control plants. Each additional column is ordered as in Fig. 2.2B based on relative C capture from the plant and is labelled with the fungal isolate name as follows: W3—Wil3, PA—P. micro A, G5—G5, M3—MW3, M6—MD06, S9—SI9, R10—R10, R4—R4, and W4—Wil4. GLC, glucose, MFS, major facilitator superfamily.

Plant and fungal gene expression in *E. grandis* roots colonized by each of the *P. microcarpus* isolates was considered and heatmaps of the expression of *E. grandis* C transporter and *P. microcarpus* C transporter and metabolism genes were generated to investigate whether fungal C acquisition was related to plant and fungal C-related activity (Fig. 5, Supplementary Table S3).

Overall, there were no strong trends between amounts of C acquired by the fungi and expression of plant C transporter genes (Fig. 5A). However, when plant gene expression patterns were compared between the Wil3 treatment and all other isolates that received lower C from the host, 13 genes of putative interest were identified. Five plant genes were more highly expressed under the Wil3 treatment as opposed to all other isolates, and eight genes were repressed. The genes showing higher expression during symbiosis encoded major facilitator superfamily transporters (*Eucgr.B02021, Eucgr.B02013, Eucgr.H01571*), an EgSWEET16 protein (*Eucgr.H04549*), and a tonoplast monosaccharide transporter (*Eucgr.I02208*). The repressed genes encoded major facilitator superfamily proteins (*Eucgr.B02309, Eucgr.B02016, Eucgr.H04438, Eucgr.H04441*), inositol transporters (*Eucgr.E03903, Eucgr.E02426*), a sugar transporter (*Eucgr.D00720*), and a sucrose transporter (*Eucgr.F00464*).

Similar to the *E. grandis* genes, the *P. microcarpus* C transporter genes also did not have strong trends with the C acquisition data (Fig. 5B). However, there were general trends between C acquisition ability of a given isolate and gene expression of the following fungal major facilitator superfamily transporters (by protein ID): Pm113068, Pm579566, Pm171041, Pm678263, Pm676370, Pm677567, Pm654975, and Pm9181. Interestingly, the majority of these *P. microcarpus* C transporters had a negative trend with C acquisition, with the exception of Pm9181 and Pm579566. Fungal C metabolism genes with general trends with the C acquisition data were also identified (i.e. higher in Wil3 as opposed to other isolates; Fig. 5C). These encoded the sugar/carbohydrate utilization enzymes fructokinase (Pm60756, Pm110989), phosphoglucose isomerase (Pm676950), and trehalase (Pm24996), and the carbohydrate biosynthesis enzymes galactinol synthase (Pm91096), glycogen synthase (Pm683186), and trehalose-6-phosphate synthase (Pm677150). Interestingly, all of these C metabolism genes were repressed in isolates that received and retained higher host C with the exception of the trehalase gene Pm24996.

### *Pisolithus microcarpus* C acquisition correlates with the plant expression of defence- and stress-related genes and regulatory genes

**Figure 6.**
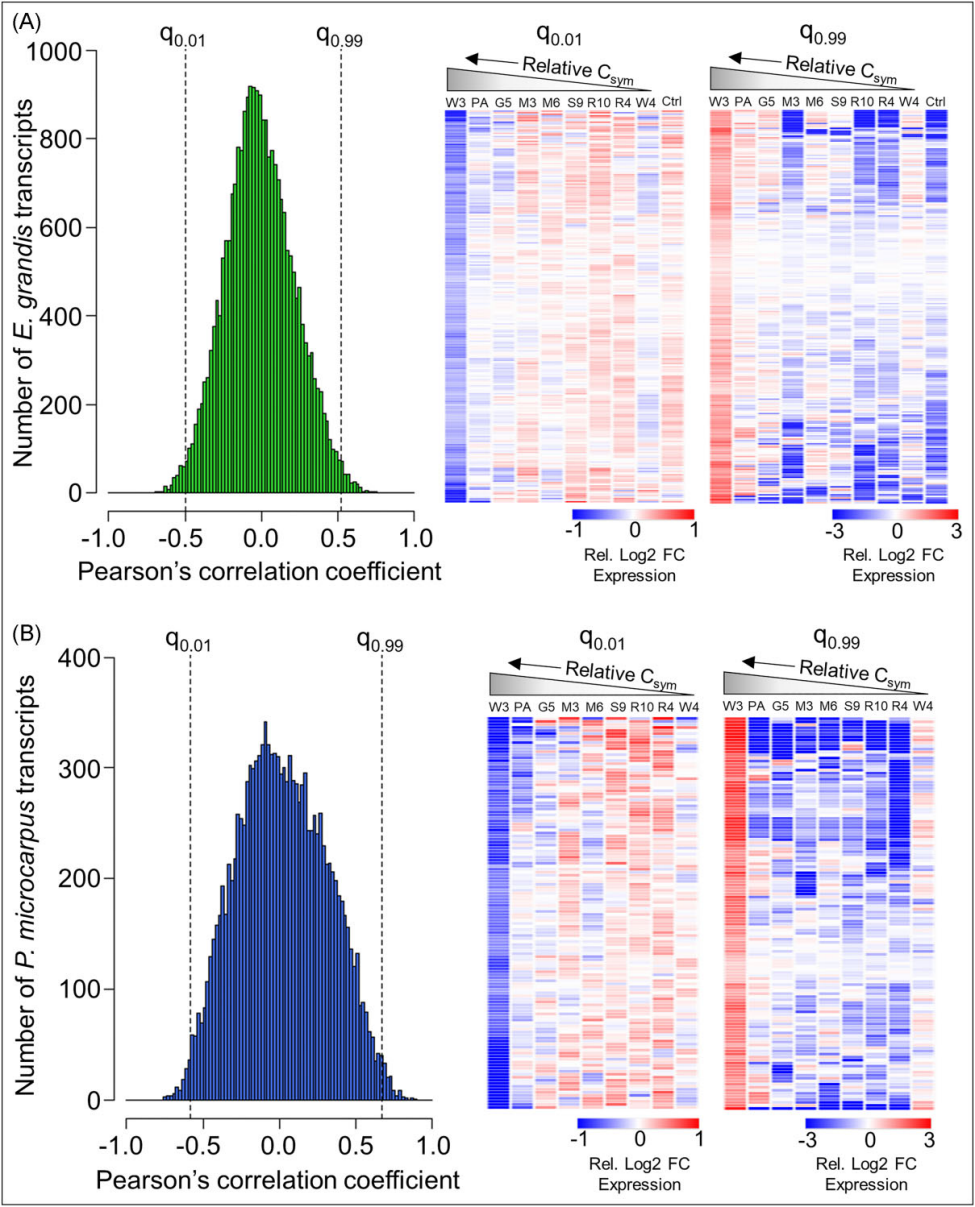
Pearson’s correlation analysis reveals gene transcripts of *E. grandis* and *P. microcarpus* correlated with fungal carbon (C) acquisition. Frequency histograms of Pearson’s correlation coefficients for correlations between C acquisition data and transcriptomic data, and heatmaps of transcripts positively (q_0.99_) and negatively (q_0.01_) correlated with C acquisition, for (A) *E. grandis*, and (B) *P. microcarpus*. Dotted lines in histograms indicate q_0.99_ and q_0.01_ of the Pearson’s correlation coefficients (i.e. where the most positively and most negatively correlated genes are located, respectively). Heatmaps display fold change expression relative to the average expression of each gene across all conditions (DESeq2-normalized and log_2_-transformed). Hierarchical clustering was performed using the Euclidean distance measure. Columns labelled as ‘Ctrl’ represent uninoculated control plants. Each additional column is ordered as in Fig. 2.2B based on relative C capture from the plant and is labelled with the fungal isolate name as follows: W3—Wil3, PA—P. micro A, G5—G5, M3—MW3, M6—MD06, S9—SI9, R10—R10, R4—R4, and W4—Wil4.

To further investigate the genes potentially involved in fungal C acquisition, a Pearson’s correlation analysis was conducted to determine the level of positive or negative correlation between host gene expression and fungal C acquisition. The top and bottom percentiles of *E. grandis* transcripts correlated with the amounts of C acquired by *P. microcarpus* fungi (i.e. the most positively correlated and most negatively correlated transcripts, respectively) were considered. In total, there were 247 transcripts each in the top and bottom percentiles of correlations with the C acquisition data (q_0.99_ Pearson = 0.52, q_0.01_ Pearson = −0.50; Fig. 6A, Supplementary Table S4).

As GO enrichment did not identify any specific pathways within these datasets, a search was conducted for genes involved in C acquisition beyond the C transporter and metabolism genes previously identified. The categories of genes searched for are presented in Table 2. 32% of all correlated genes (26% of positively correlated and 39% of negatively correlated genes) related to growth/cell cycle and/or transcription regulation, including genes for methyltransferases, pentatricopeptide repeat proteins, tetratricopeptide repeat proteins, F-box superfamily proteins, ubiquitin-associated proteins and zinc fingers (Table 2). Genes related to defence/disease resistance and/or stress response accounted for 20% of correlated genes (22% of positively correlated and 19% of negatively correlated genes), including genes for laccases, U-box superfamily proteins, and LRR domain and NB-ARC domain disease resistance genes. Carbon metabolism genes made up <3% of all correlated genes.

**Table 2.** Predicted carbon (C)-related functions of *E. grandis* genes positively and negatively correlated with amount of C acquired by the fungi. Correlated genes were identified via Pearson’s correlation analysis of DESeq2-normalized genes.

C-related functional category	% of positively correlated genes	% of negatively correlated genes	% of correlated genes
Carbohydrate biosynthesis	1	0	1
Defence/disease resistance	12	9	10
Growth/cell cycle regulation	11	15	13
Host–fungus interaction	1	2	1
Sugar/carbohydrate catabolism	3	1	2
Signal transduction	9	7	8
Stress response	10	10	10
Transcription regulation	15	24	19
Transporter	5	3	4

### *Pisolithus microcarpus* C acquisition negatively correlates with fungal expression of signalling, regulatory, and defence genes

Gene expression in the *P. microcarpus* isolates correlated to symbiotic C acquisition with *E. grandis* was also analysed via Pearson’s correlation analysis. There were 130 transcripts each in the top and bottom percentiles of correlations with the C acquisition data (q_0.99_ Pearson = 0.67, q_0.01_ Pearson = −0.58; Fig. 6B, Supplementary Table S5). Similar to the analysis of *E. grandis* gene correlation, within this data set *P. microcarpus* genes relating to C acquisition were targeted (Table 3). Only nine of the positively correlated genes fell into these categories, of which six were related to transcription regulation and none were related to C metabolism. Negatively correlated genes were largely annotated as being involved in signal transduction, transcription regulation, growth/cell cycle regulation, and defence/disease resistance. Three carbohydrate biosynthesis genes and seven sugar/C catabolism genes were also identified as negatively correlated, of which one was related to inositol metabolism.

**Table 3.** Predicted carbon (C)-related functions of *P. microcarpus* genes positively and negatively correlated with amount of C acquired from the host plant. Correlated genes were identified via Pearson’s correlation analysis of DESeq2-normalized genes.

C-related functional category	% of positively correlated genes	% of negatively correlated genes	% of correlated genes
Carbohydrate biosynthesis	0	3	2
Defence/disease resistance	0	15	7
Growth/cell cycle regulation	1	16	8
Host–fungus interaction	0	8	4
Sugar/carbohydrate catabolism	0	6	3
Signal transduction	2	21	11
Stress response	1	6	1
Transcription regulation	5	19	12
Transporter	1	6	3

## Discussion

While knowledge of the factors that affect C acquisition by ECM fungi across distantly related lineages have improved in recent years, understanding of how this is affected by genetic variability between closely related fungi is less well characterized. In this study, as hypothesized, the *P. microcarpus* isolates tested varied in the amounts of C acquired from *E. grandis*. This variation between the fungi allowed us to uncover physiological and genetic mechanisms that correlate with host-to-fungus C exchange.

Early reports considering C allocation to mycorrhizal root tips found that fine roots colonized by ECM fungi received between 5–18× more photosynthate than uncolonized roots (Cairney et al. 1989, Wu et al. 2002). A significant portion of this C is then transferred into the ERM within the first few days (Wu et al. 2002). For this reason, it was originally hypothesized that isolates of *P. microcarpus* with higher amounts of host-derived C acquired would also show higher levels of root tip colonization. Congruent with this theoretical framework, a study by Shinde et al. (2018) found that ECM-colonized *Populus tremuloides* plants under nonlimiting nutrient conditions had higher levels of certain carbohydrates in their colonized roots, including starch and sucrose. Furthermore, plants colonized by *Paxillus involutus*, which had up to 13% higher colonization rates than *L. bicolor*, had higher levels of starch in their fine roots than *L. bicolor*-colonized plants. However, the results of our current study demonstrate a negative correlation between C acquisition and colonization rates of the host root system. Findings by Hortal et al. (2017), who measured both colonization rate and symbiotic C content in the mycelia of three *Pisolithus* isolates associated with *E. grandis*, also found that colonization rate was not associated with host-derived C. Further studies examining both plant root colonization and C transfer from host to fungus are needed to gain a better understanding of the importance of root colonization rate in C acquisition and whether there are ECM fungal genera that require high rates of root colonization to obtain host C while others do not.

Our aim to identify host genes putatively correlated to greater C acquisition by an ECM fungus identified that 20% of genes correlated with this trait were involved in defence or stress responses. While plant defence and stress responses to colonization by mycorrhizal fungi have been reported previously, they are often observed in the context of symbiosis formation or defence priming rather than the control of C transfer to symbionts (Pozo and Azcón-Aguilar 2007, Kiers et al. 2011, Garcia et al. 2015, Plett et al. 2015b, Kanekar et al. 2018, Watts-Williams et al. 2018, Dreischhoff et al. 2020). However, plants are known to employ defence mechanisms against root-dwelling fungi to limit resource loss and to prevent mycorrhizal fungi from becoming parasitic towards their hosts (Ferreira et al. 2007, Agren et al. 2019). Supporting this, Hortal et al. (2017) found elevated host defence gene transcription and reduced colonization of a *P. microcarpus* isolate that provided less N than its competitors. Similarly, the induction of several defence-related pathways in late stage of symbiosis establishment in *Populus*–*L. bicolor* ECM mycorrhizal root tips was suggested to be a means of host control to curtail over-colonization by the fungal partner (Plett et al. 2014). A secondary reason for the involvement of defence pathways in colonized roots could be due to direct induction of defences by sugar molecules themselves. Several pathogens have been found that manipulate plant hosts to redirect sugar into infected tissues (Chen et al. 2010, Chong et al. 2014). The perception of glucose molecules in these situations then leads to induction of innate cell immunity (Baena-Gonzalez 2010, Rampitsch and Bykova 2012, Guo et al. 2013). Therefore, defence- and stress-related gene transcription may be correlated to C presence and transfer at the symbiotic interface, not because these pathways benefit the ECM fungus, but because the plant is preventing C drainage to ECM fungi. This may also provide an explanation as to why a negative correlation between C acquisition and colonization rate was observed in our study as the plant host may be actively reducing colonization of the more C-demanding fungi. Furthermore, a positive correlation was found between C acquisition and the depth of the Hartig net, which may suggest that fungi that can penetrate further into the root tip to receive C are better able to tolerate plant defence mechanisms. The role of plant defence and stress mechanisms in controlling colonization and C acquisition by ECM fungi should be further investigated. Also, future studies on mechanisms behind C transfer in ECM symbioses should take into account that function in mycorrhizal symbioses is highly complex and can be influenced at various levels other than transcriptional regulation, including protein localization and activity.

The exploration type of fungal mycelium, which describes the rate and form of a given ECM fungal species to grow through soil (Agerer 2001), may drive levels of C acquired from a host. Based on one species, *Pisolithus* hyphae are classified as a long-distance exploration type, defined as having sparser but further-reaching mycelium (Agerer 2001). Not only does the current study demonstrate that this may be an over-simplification, as mycelial growth form varied significantly between the tested isolates of *P. microcarpus*, host C in fungal tissues was found not to be positively correlated with radial mycelial growth rate or thickness of the fungal mantle surrounding plant root tips, but rather with density of fungal mycelium and Hartig net depth. This implies that hyphal branching and a dense mycelium growth pattern might be a better predictor of high C acquisition than extension of mycelia into the substrate. Using a microcosm-based study system, Wu et al. (2002) found similar results whereby ECM fungi with denser hyphal growth concentrated higher portions of host C. This would support the hypothesis that one mechanism by which ECM fungi acquire C from their host is due to a greater sink strength created by fungal biomass, although the signalling connecting growth characteristics to C acquisition remain to be investigated in future studies. While such research questions are typically hard to measure in natural systems, it is known that host C accumulates most in areas with high fungal biomass (Leake et al. 2001). Furthermore, when C is nonlimiting to the host (e.g. due to elevated CO_2_, during summer), ECM species with faster growth and thicker mantles around colonized roots are favoured, while the converse is true when the host is C-limited (e.g. due to defoliation, during winter; Godbold et al. 1997, Markkola et al. 2004, Parrent and Vilgalys 2007, Pritchard et al. 2008, Saravesi et al. 2008). These previous results would argue that, in addition to the findings of this study regarding fungal C demand, the C status of the host is also a driver of photosynthate allocation to ECM partners. This raises the question of whether a high amount of host-delivered C causes high fungal biomass production or if inherent growth properties of the fungi result in C supply by the host. ECM C transfer is likely to be both a cause and consequence of fungal biomass production, as well as of plant nutrient requirements and fungal ability to acquire and deliver these nutrients from soil.

The likelihood of an ECM fungus to form a C sink may depend on its ability not only to acquire C from its host but also to rapidly metabolise C into various intermediate storage compounds, including trehalose and glycogen (López et al. 2007, Wiemken 2007, Nehls 2008). Genomic and transcriptomic studies have indicated the abilities of several ECM fungal species, such as *L. bicolor, Tuber melanosporum*, and *A. muscaria*, to both utilize sugars and synthesize C storage compounds during symbiosis (Deveau et al. 2008, Nehls 2008, Ceccaroli et al. 2011). Plett et al. (2021) found that when growing under the same glucose availability as used in the current study (0.01% w/v), *P. microcarpus* isolates MW3, SI9, R10, and R4 all had little to no glucose in the free sugar compositions of their mycelia, and trehalose was instead the predominant storage sugar. The analysis of fungal C metabolism gene expression in this study revealed that *P. microcarpus* fungi with significantly different C acquisition values had different expression levels of enzymes for the utilization of sugars, such as fructose and glucose, and for the biosynthesis of the carbohydrates trehalose, glycogen, galactinol, and inositol. However, expression was not necessarily higher in the fungi with higher C values and Pearson’s correlation analysis of fungal C metabolism genes did not find genes significantly positively correlated with C acquisition. It should be noted that neither the study by Plett et al. (2021) nor the current study examined C acquisition within colonized plant root tips, which are the actual site of C exchange between plants and ECM fungi, but rather ERM. Further work combining the stable isotope and sugar composition analyses from the two studies, to confirm that host-acquired glucose is converted to C storage compounds at the Hartig net within colonized root tips, would provide greater understanding of the potential mechanisms of C sink formation by ECM fungi.

Together, the findings of this study suggest that ECM fungal acquisition of host-derived C involves both fungal and plant mechanisms. This study provides early indications that C acquisition may be highly variable within a given species of ECM fungi, whereby this trait is dependent on individual C demands as determined by the ability of the fungus to metabolize host-derived C and convert it into biomass. Our study also demonstrates that C acquisition is not purely under the control of the fungus, but also depends on the ability of the host plant to reduce colonization and, therefore, C drain. Advances in current understanding of the molecular mechanisms behind the exchange of C within the ECM symbiosis will be essential in better understanding the roles of this plant–fungus association in the C cycling of temperate and boreal forest ecosystems and, thus, in the global terrestrial C sink.

## Supplementary Material

fiad037_Supplemental_Files
