# Supplementary material for: Acquisition of host-derived carbon in biomass of the ectomycorrhizal fungus Pisolithus microcarpus is correlated to fungal carbon demand and plant defences
Source: FEMS Microbiol Ecol. 2023 Mar 31;99(5):fiad037. doi: 10.1093/femsec/fiad037 (PMC10191194; doi:10.1093/femsec/fiad037)
Supplement: fiad037_Supplemental_Files [file fiad037_supplemental_files.zip › Supp_data_captions.docx]

Supplementary information captions

**Table S1 Collection sites of *Pisolithus microcarpus* isolates.** Data obtained from Keniry (2015) and Jourand et al. (2010).

**Table S2 ITS sequences of fungal isolates for phylogenetic analysis.** Sequences were obtained from National Center for Biotechnology Information (NCBI) Nucleotide database (https://www.ncbi.nlm.nih.gov/nuccore) or were sequenced at the Hawkesbury Institute for the Environment (HIE).

**Table S3 Expression values of *Eucalyptus grandis* carbon (C) transporter genes, *Pisolithus microcarpus* C transporter genes and *P. microcarpus* C metabolism genes.** Values represent the average, fold change expression values (DESeq2-normalised, log2-transformed) of three replicates of *E. grandis* or *P. microcarpus* during symbiosis. MFS, major facilitator superfamily, P, phosphate.

**Table S4 Functional annotations and gene expression count values of *Eucalyptus grandis* genes positively and negatively correlated with amount of C acquired by the fungi.** Count values displayed are the average of three replicates (DESeq2-normalised). DR, disease resistance, MFS, major facilitator superfamily, PCC, Pearson’s correlation coefficient.

**Table S5 Functional annotations and gene expression count values of *Pisolithus microcarpus* genes positively and negatively correlated with amount of C acquired by the fungi.** Count values displayed are the average of three replicates (DESeq2-normalised). PCC, Pearson’s correlation coefficient, DR, disease resistance.
